# Image processing for cryogenic transmission electron microscopy of symmetry-mismatched complexes

**DOI:** 10.1042/BSR20170203

**Published:** 2018-03-16

**Authors:** Juha T. Huiskonen

**Affiliations:** 1 Helsinki Institute of Life Science and Molecular and Integrative Biosciences Research Program, Faculty of Biological and Environmental Sciences, Viikinkaari 1, University of Helsinki, 00014 Helsinki, Finland; 2 Division of Structural Biology, Wellcome Centre for Human Genetics, Roosevelt Drive, University of Oxford, OX3 7BN Oxford, UK

**Keywords:** asymmetric reconstruction, Cryo-EM, expanding symmetry, focused classification and refinement, localized reconstruction, relaxing symmetry, symmetry mismatch

## Abstract

Cryogenic transmission electron microscopy (cryo-TEM) is a high-resolution biological imaging method, whereby biological samples, such as purified proteins, macromolecular complexes, viral particles, organelles and cells, are embedded in vitreous ice preserving their native structures. Due to sensitivity of biological materials to the electron beam of the microscope, only relatively low electron doses can be applied during imaging. As a result, the signal arising from the structure of interest is overpowered by noise in the images. To increase the signal-to-noise ratio, different image processing-based strategies that aim at coherent averaging of signal have been devised. In such strategies, images are generally assumed to arise from multiple identical copies of the structure. Prior to averaging, the images must be grouped according to the view of the structure they represent and images representing the same view must be simultaneously aligned relatively to each other. For computational reconstruction of the 3D structure, images must contain different views of the original structure. Structures with multiple symmetry-related substructures are advantageous in averaging approaches because each image provides multiple views of the substructures. However, the symmetry assumption may be valid for only parts of the structure, leading to incoherent averaging of the other parts. Several image processing approaches have been adapted to tackle symmetry-mismatched substructures with increasing success. Such structures are ubiquitous in nature and further computational method development is needed to understanding their biological functions.

## Symmetry mismatches in biological macromolecular complexes

### Deviations from perfect symmetry limit the attainable resolution in cryo-TEM

Cryogenic transmission electron microscopy (cryo-TEM) allows reconstructing 3D density maps of biological macromolecules from projection images of individual copies of the macromolecule, referred to as single particles [1]. A prerequisite for attaining high resolution in the reconstructed density map is coherent averaging of a sufficient number of single particle images representing different views of the original structure. This coherency is limited, among other factors, by deviations of the individual particles from the symmetry of the idealized structure.

Symmetry mismatches are ubiquitous in biological macromolecular complexes and pose several problems to their structural analysis. Firstly, conformational differences in the subunits making the complex [2] and intrinsic flexibility in the interactions between the subunits [3–5] break the symmetry assumption and thus limit the attainable resolution if symmetry is imposed in the reconstruction process. Secondly, sub-stoichiometric binding, or variable occupancy, of some of the substructures leads to partial occupancy in the averaged density map [6]. Thirdly, mismatch in the symmetry of a substructure and its binding region in the larger structure [6–12] may lead to incorrect density for the substructure.

In this review, I will discuss cryo-TEM image processing methods that aim at retaining coherent averaging of symmetry-mismatches complexes. I will start by defining a nomenclature to describe various types of symmetry mismatches. I will then discuss several types of challenging structures and review image processing approaches that have been used to resolve their symmetry mismatches. Finally, I will give several examples on recent success stories and discuss directions for future method development.

### Nomenclature to describe symmetry mismatches

**Figure 1:**
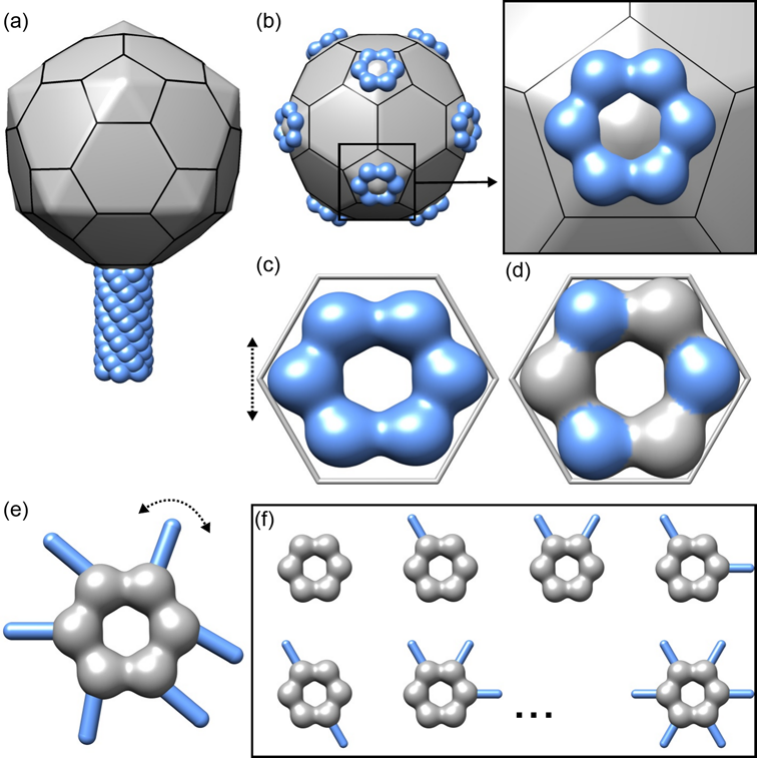
Different types of symmetry mismatches in macromolecular complexes (**a**) A schematic model of an icosahedrally symmetric particle (gray) with a symmetry-mismatched tail with C6 symmetry (blue) bound to one of the five-fold vertices of the particle (one I-C5–C6 symmetry mismatch). (**b**) An icosahedrally symmetric particle with 12 hexamers (C6 symmetry) bound to each of the 12 five-fold vertices (12 I-C5–C6 symmetry mismatches). The inset shows a close-up of the vertex outlined with a box. Coloring as in (a). (**c**) A model for a quasi-symmetric hexamer (blue; *q*C6 symmetry mismatch). An equilateral hexagon (gray) is shown for a reference. The direction of distortion is indicated with an double arrow. (**d**) A model for a pseudo-symmetric hexamer (*p*C6 symmetry mismatch). Despite perfect six-fold shape at low resolution, three of the subunits are different in their sequence (blue) from the rest of the subunits (gray), illustrated here with different colors. (**e**) A model for a hexameric structure (gray) where each of the subunits binds a flexible appendix (blue; six C6-C1–*f*C1 symmetry mismatches). The direction of flexibility in the appendices is indicated with a double arrow. (**f**) Illustration of hexamers with appendices manifesting variable occupancy (a population of particles with C6-C1–*v*C1 symmetry mismatches). The fact that not all possible occupancy states are shown is indicated with three dots.

To discuss different and often complex symmetry mismatches, we define the following nomenclature (Figure 1). First we denote the dominant symmetry of the structure, namely cyclic (Cn), dihedral (Dn), tetrahedral (T), octahedral (O) or icosahedral (I). Next, in the cases where the structure has a local symmetry axis at the binding site of the symmetry-mismatched substructure, this is denoted. For example, if the site of the symmetry mismatch is at the five-fold symmetric vertex of an icosahedrally symmetric particle, we shall define this as ‘I-C5’. (For consistency, if no local symmetry is present at the binding site, we denote this with C1.) Finally, we define the symmetry of the symmetry-mismatched substructure. For example, a hexameric substructure binding to a five-fold symmetric vertex of an icosahedrally symmetric particle is denoted as ‘I-C5–C6’ (Figure 1a). Often more than one symmetry mismatch may exist. In our example, there could be up to 12 symmetry mismatches of the type I-C5–C6, since there are 12 C5 symmetry axes in a structure with icosahedral symmetry (Figure 1b).

The structure as a whole may exhibit flexibility or contain pseudo-symmetric components, breaking its apparent dominant symmetry. For example, we refer to a hexamer with inherent flexibility as quasi-hexameric symmetry and denote it as ‘*q*C6’ (Figure 1c). By pseudo-symmetry (*p*), in contrast, we refer to structures that can be considered to be symmetric at low resolution, but this symmetry assumption breaks down at high resolution, for example if the amino acid sequences of the symmetry-related subunits are not identical (Figure 1d). In such cases, it is often more informative to refer to the structure having a certain pseudo-symmetry rather than no symmetry at all.

Also flexibility (*f*) of the substructure may cause or further contribute to the symmetry mismatch in the complex. For example, a hexamer that has six asymmetric substructures, each binding an asymmetric unit in a flexible manner, can be referred to as ‘C6-C1–*f*C1’ (Figure 1e). Furthermore, in such cases, it is possible that not all of the possible sites are occupied by the substructure. In other words, the substructures may display variable occupancy (*v*). In our example, if the substructure is binding at some but not necessarily all of the subunits of the hexamer, the complexes form a population of ‘C6-C1–*v*C1’ particles with different occupancy states (Figure 1f).

### Structures with one symmetry-mismatched substructure

Symmetry mismatches are ubiquitous in biological macromolecular complexes. We start by discussing cases where a single substructure has a different symmetry than the rest of the structure (Figure 1a). Bacterial secretion systems [13], tails of many bacteriophages [7,8,11], and genomes of isometric viruses [6,10,14,15] are prime exemplars of this type of symmetry mismatch. The type III secretion system of the bacterial species *Salmonella typhimurium* is characterized by a C24–C15 symmetry mismatch between the inner (C24) and outer (C15) rings of the needle complex [13]. The portal vertex (C5) of T7 bacteriophage has two substructures, the portal itself (C12) and the core stack (C8) [8], constituting I-C5–C12 and I-C5–C8 symmetry mismatches, respectively. Finally, GroEL is a barrel-shaped macromolecular complex with D7 symmetry. This complex acts as a chaperonin assisting folding of asymmetric polypeptide chain substrates [16], constituting a D7–C1 symmetry mismatch between the chaperonin and the substrate.

### Structures with several symmetry-mismatched substructures

Instead of a single symmetry-mismatched substructure, several of such substructures may exist in one particle (Figure 1b). Many viruses with icosahedrally symmetric capsids have ‘turrets’ of ‘fibers’ bound to each of the 12 five-fold vertexes of the capsid [17,18]. In many cases the symmetry of these substructures deviates from the local C5 symmetry of the vertex. Adenovirus fibers are trimers binding to the five-fold vertices of the icosahedrally symmetric capsid (I-C5–C3 symmetry mismatch) [17,19]. Cystoviruses are bacteriophages that use hexameric packaging NTPases for translocating RNA segments into preformed icosahedrally symmetric capsids. These hexamers create a I-C5–C6 symmetry mismatch at each of the 12 vertices and their structures cannot be resolved with icosahedral reconstruction [12,18].

### Symmetric structures with intrinsic flexibility

Many ring-shaped structures are built from several asymmetric units that are arranged following cyclic (Cn) symmetry. Sometimes deformations from the perfect ring-shape lead to a mismatch from perfect symmetry, or quasi-symmetry (*q*Cn; Figure 1c). For example, the nuclear pore complex (NPC) is built from eight asymmetric units, which follow approximate C8 symmetry. However, deviations from perfect C8 symmetry (*q*C8 symmetry) limit the resolution of reconstructions calculated by averaging tomographic sub-volumes of whole NPCs [3].

Spherical structures follow often octahedral (O) or icosahedral (I) symmetry and deviations from perfect spherical shape lead to quasi-symmetry. COPII cages are shaped as cuboctahedrons and follow octahedral symmetry [20,21]. However the cages are flexible [4] and their symmetry can be defined as *q*O. Icosahedrally symmetric capsids of large viruses are also often flexible (*q*I symmetry) [22,23]. Quasi-symmetry unavoidably limits resolution of cryo-TEM reconstructions when symmetry is applied.

It is worth noting that all symmetric structures have some flexibility and thus quasi-symmetry. It may be useful to describe a structure having quasi-symmetry only if relaxing this symmetry assumption has made a difference at the resolution relevant to the discussion at hand.

### Structures with pseudo-symmetry

Many large complexes consist of subunits that are homologous and may be thus very similar in their fold, but differ in their sequence, leading to pseudo-symmetry (Figure 1d). For example, the TRiC chaperonin complex consists of two eight-fold rings of subunits facing each other and the whole complex thus approximates D8 symmetry [24]. As each of the eight subunits has a different sequence, however, the rings have pseudo eight-fold symmetry and the structure as a whole can be described as *p*D8. This pseudo-symmetry has lead to ambiguity in the arrangement of the eight subunits [24–26].

### Substructures with flexible binding

Interactions between the structure and its substructures may be flexible (*f*; Figure 1e). For example, viruses with icosahedrally symmetric capsids may bind up to 60 subunits in a flexible way (I-C1–*f*C1). This is the case in picornaviruses that bind up to 60 integrin receptor ectodomains in a flexible manner [5]. This type of symmetry mismatch results in incoherent averaging of the integrins if icosahedral symmetry is applied [5,27]. In the simplest possible case for this class of symmetry mismatches, we consider a complex with two asymmetric parts that can move or rotate relative to each other. A prime exemplar of such a complex is the ribosome, where the large and small subunits undergo a ratcheting motion relative to each other [28]. For completeness, we define this special case as ‘C1–*f*C1’ symmetry mismatch.

### Substructures with variable occupancy

Many macromolecular complexes bind other components in sub-stoichiometric amounts, leading to variable occupancy (*v*), also referred to as compositional heterogeneity (Figure 1f) [29]. This is common for example when purified Fabs [30] or soluble receptor fragments [5] are bound to viral capsids to resolve their mode of interaction by cryo-TEM. If such particles with variable occupancy are averaged together and symmetry is applied, the averaged density of the substructure will show partial occupancy in the density map (also referred to as partial density).

## Methods to deal with symmetry-mismatched structures

Cryo-TEM, when combined with single particle averaging, allows analyzing and resolving symmetry mismatches. For coherent averaging of single particles, the orientation (often defined as a triplet of Euler angles) and origin (two or three coordinates for 2D and 3D single particles, respectively) are first determined [31]. In template-based methods, a model structure (or a set of 2D projections calculated from it) is used to determine these parameters for all of the observed particles. The orientation and origin search is iterated and after each iteration a new template structure is reconstructed. This iterative process is referred to as refinement of the particles and is typically run until no changes in the orientation and origin parameters are observed. Standard template-based single particle refinement methods have been extended to allow dealing with symmetry mismatches. This is true both for 2D and 3D single particle refinement methods, the latter of which is often referred to as sub-tomogram of sub-volume averaging [32]. Below I focus on extensions of these template-based refinement methods in the context of 2D single particles to resolve structures of symmetry-mismatched structures.

### Standard asymmetric refinement

Structures that have exactly one symmetry-mismatched component (Figure 1a) or pseudo-symmetry (Figure 1d) can be addressed by standard asymmetric refinement [11]. If the single symmetry-mismatched component, such as the viral genome, is locked always the same way relative to the symmetric component, such as the icosahedrally symmetric capsid, each particle can be assumed to be identical with one another, and such a structure is amenable to single particle averaging. Standard asymmetric refinement relies on conventional single-particle refinement, where the orientation parameters are searched for over the full orientation space at each iteration. A model of the particle can first be calculated imposing the dominant symmetry and this model can then serve as a starting model for the asymmetric refinement of the particles. Downside of the standard asymmetric refinement is that orientations determined often first using the dominant symmetry are not utilized and search over the full angular space is computationally expensive.

**Figure 2:**
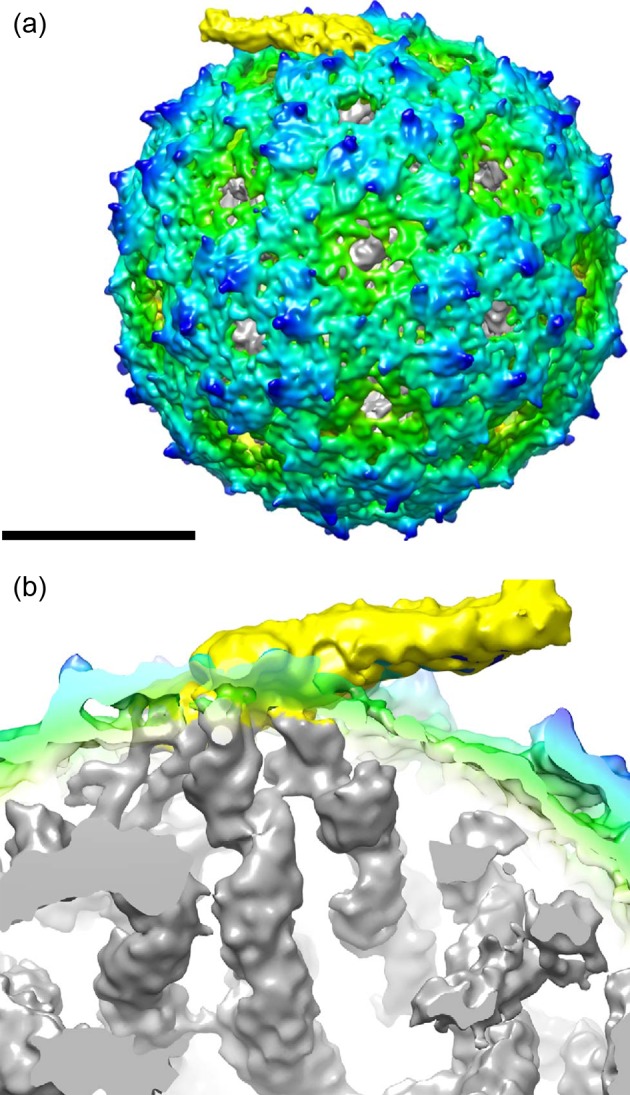
Asymmetric reconstruction of bacteriophage MS2 (**a**) Radially depth cued isosurface representation of the asymmetric reconstruction of MS2 virion. Density has been colored from gray to green to cyan to blue to yellow, in the order of small to large radius. Scale bar, 100 Å. (**b**) A cut open view of the density under the A-protein (yellow). Ordered RNA density is in gray. Figure reproduced from [15].

Despite its limitations this method has recently been applied successfully to solve the structures of many viral genomes (Table 1), for example that of the bacteriophage MS2 [15]. The authors of this study first applied icosahedral symmetry during refinement. The resulting icosahedrally symmetric model was then used as a starting model for asymmetric refinement, which resolved the single-strand RNA genome of the virus, in addition to the symmetry-mismatched A-protein (I–C1 symmetry mismatch; Figure 2).

**Table 1:** Some examples of symmetry-mismatched structures published in the past 3 years.

Complex	Basis structure	Binding site	Substructure	Sym. mism.	*N*	Method^*a*^	Sign. sub.?	Ref.
Adenovirus D26 vertex	Capsid (I)	Vertex (C5)	Fiber (C3)	I-C5–*f*C3	12	LocalRec	No	[19]
Bacteriophage phi6 vertex	Capsid (I)	Vertex (C5)	Hexamer (C6)	I-C5–C6	12	LocalRec	Yes	[12]
Cypovirus genome	Capsid (I)	N/A	Genome (*p*D3)	I–*p*D3	1	SymRelax	Yes	[6]
Cypovirus genome	Capsid (I)	N/A	Genome (*p*D3)	I–*p*D3	1	SAR	Yes	[10]
GroEL	GroEL (D7)	N/A	N/A	*q*D7	14	LocalRec	Yes	[2]
Apoptosome	Central hub (C7)	Hub subunit (C1)	Sickle-like density (C1)	C7-C1–*v*C1	7	SymExpand/FocusedClass	Yes	[33]
COPII	COPII cage (O)	N/A	N/A	*q*O	12	LocalRec	No	[4]
Bacteriophage phi6 polymerase	Capsid (I)	Face (C3)	Polymerase (C1)	I-C3–*v*C1	20	LocalRec	Yes	[4]
HIV-1–intasome	IN dimer (C2)	Core (C1)	Flanking subunit (C1)	C2-C1–*f*C1	2	SymExpand/FocusedClass	No	[34]
FMDV–receptor complex	Capsid (I)	RGD loop (C1)	Integrin receptor	I-C1–*vf*C1	60	LocalRec	Yes	[5]
MS2 genome	Capsid (I)	N/A	Genome	I–C1	1	SAR	No	[15]
Qβ genome	Capsid (I)	N/A	Genome	I–C1	1	SAR	No	[14]
Rift Valley fever virus	Capsid (I)	N/A	N/A	*q*I	12 or 60	LocalRec	No	[35]

a The following abbreviations for the methods are used: LocalRec (sub-particle based localized reconstruction methods), SymRelax (symmetry relaxation methods), SAR (standard asymmetric reconstruction), and SymExpand/FocusedClass (symmetry expansion combined with focused classification).

### Asymmetric refinement and reconstruction by relaxing symmetry

To speed up the asymmetric refinements in cases such as MS2 described above, searching the full orientation space is often not necessary [7,11]. Instead, it is possible to limit the search based on the dominant, original symmetry group. For example, in the case of an icosahedrally symmetric capsid enclosing an ordered genome, it is sufficient to check which of the 60 views of the template (the original view and the 59 symmetry-related views in the icosahedral point group) matches the observed particle the best [6]. The best view parameters are defined for all of the particles in asymmetric reconstruction and the process is iterated. This is referred to as ‘symmetry relaxation’ because higher symmetry assumption is relaxed (in this example from I to C1) [11]. The refinement process is otherwise similar to standard asymmetric refinement, the only difference is the way the relatively small set of discrete orientations to be tested is selected.

Symmetry relaxation/asymmetric refinement method has recently been used to obtain the structure of the cypovirus genome, revealing the non-spooled organization of the ten genome segments with the bound RNA-dependent RNA polymerases (Table 1) [6]. Like all virus genomes, the genomes of cypoviruses are also strictly speaking asymmetric, however, in these viruses the genome segments create an ordered arrangement approximating D3 symmetry (*p*D3) inside icosahedrally symmetric capsids (I–*p*D3 symmetry mismatch; note that as there are 10, not 12, genome segments and associated polymerases, this *p*D3 symmetry has also incomplete occupancy). Similar results were obtained with an ad hoc approach, where the orientations from an asymmetric refinement were tested against the 60 equivalent orientations of the icosahedral particle and those that were in consistent agreement were used to reconstruct the genome [10].

### Focused refinement of symmetry-mismatched structures

**Figure 3:**
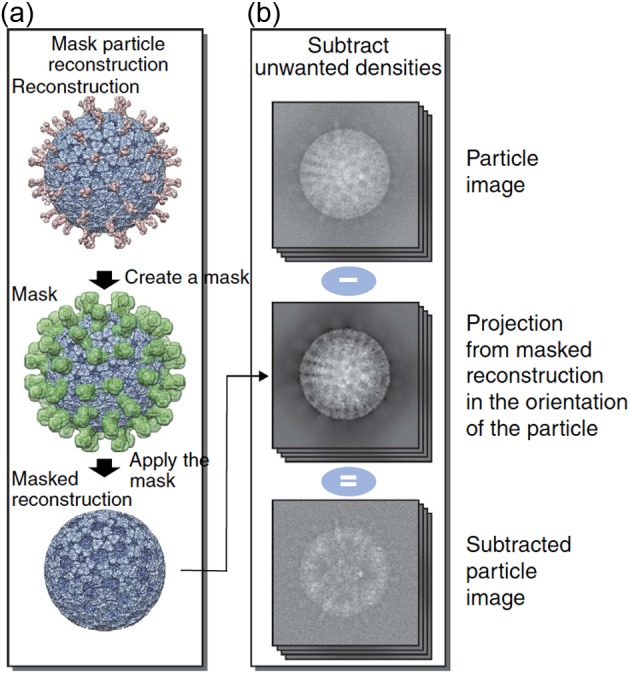
Partial signal subtraction (**a**) Subtraction of unwanted densities is illustrated using the rotavirus particle as an example. To subtract the virus capsid (blue) to allow analysis of the spikes (red), first a mask (green) corresponding to the spike densities is defined. A masked reconstruction, where the spikes have been removed, is calculated. (**b**) Computational projections of the masked reconstruction are subtracted from the experimental images of the particles. This results in images that contain contribution from the spikes only. These images can then be used to analyze the structure of the spikes without interference from the capsid. Figure reproduced from [4].

A common problem with the standard asymmetric refinement method is that often the signal from the symmetric part of the structure dominates the alignment, and the orientation parameters of the symmetry-mismatched substructure are thus not determined with high enough fidelity to resolve its structure correctly [11,36]. To improve the sensitivity of these methods, refinement can be focused on the symmetry-mismatched substructure, such as the cypovirus genome given as an example above [6,10]. This is achieved by subtracting the signal arising from the symmetric part of the 3D template structure used during refinement, and ideally also in the 2D projection images of the particles. Removing the unwanted signal in the 3D template can be achieved by a simple masking operation by providing a binary mask to be used during the refinement. Removing the unwanted signal in the 2D projection images can be achieved by partial signal subtraction (Figure 3) [6,9,10,36–38]. After symmetry relaxation and asymmetric refinement of the symmetry-mismatched structure, the determined orientation parameters can be used to calculate a composite structure from the original images, from which no signal has been subtracted. This structure, calculated without symmetry, can in theory reveal the connections between symmetry-mismatched components [12,19].

### Expanding symmetry for focused refinement of several substructures

Above, we have limited our discussion to structures that have only one symmetry-mismatched substructure. If there is more than one such substructure (Figure 1b) or quasi-symmetric subunits (Figure 1c), they can all be subjected to focused refinement (with or without classification) protocols after expanding the symmetry of the particle [39,40]. For each particle, all orientation parameters that are equivalent in the original symmetry group are calculated. After this symmetry expansion, each particle image is effectively used several times in the refinement, but in each case a different symmetry operator is chosen to ensure that a unique substructure is considered and aligned using signal from the template in the area defined by a 3D mask [39,40].

### Localized reconstruction of symmetry-mismatched substructures

While symmetry expansion allows dealing with more than one symmetry-mismatched substructure per particle, it is worth noting that this approach is most suitable for local refinements of relatively small deviations in the origins and orientations of the substructures, because the orientation searches are carried out around the center of mass of the whole complex, not that of the substructure itself [37]. Furthermore, with large particles and high symmetry, computational costs after symmetry expansion may become prohibitive in practice [4]. For example, in the case of viruses with icosahedrally symmetric capsids, all particle images, which are often large in their dimensions, would be considered 60 times in each refinement and classification step.

**Figure 4:**
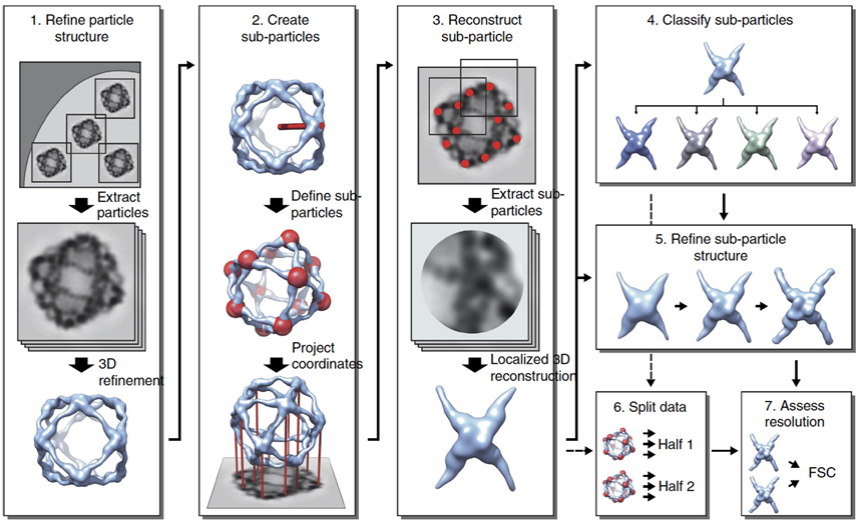
Typical workflow for localized reconstruction Schematic diagram of the workflow for localized reconstruction. First the structure of the macromolecular complex is solved using conventional 3D refinement (1), after which the locations of the substructures (red spheres) are calculated based on the particle orientation, a symmetry operator and a vector defining one substructure relative to the particle model (red stick; 2). After extracting the sub-particles (red dots) from the particle images, a localized 3D reconstruction is calculated (3). This reconstruction can be used as a starting model for further classification (4) and 3D refinement (5) of sub-particles to improve the structure. Finally two independent sets of data (6) are compared by Fourier shell correlation (FSC) to assess the resolution of the reconstruction (7). Figure and legend reproduced from [4].

To overcome these limitations, methods utilizing the concept of ‘sub-particle’ have been developed [4,7,9,32,37,41,42]. In these methods, the original image of each particle is reboxed into smaller areas, or ‘sub-particles’, each corresponding to a projection of the substructure of interest (Figure 4). The alignment parameters (Euler angles and origins) of the sub-particles can be calculated from those of the original particles, position of the substructure relative to the 3D model of the particle, and the symmetry operators [4]. The sub-particles can then be extracted from the original particle images (optionally after partial signal subtraction), centered around the origin of the sub-particle. Instead of the particle images, the sub-particles can also be extracted from the original micrographs [4,37]. The sub-particles and their alignment parameters can then be used to calculate a 3D reconstruction of the substructure. It is worth noting that the height of the sub-particle relative to the particle origin can be taken into account by adjusting the average defocus parameter of the particle and then used for accurate contrast transfer function correction, effectively taking the thickness of the sample into account [4].

Normal single particle averaging workflows can be used to improve the resolution of the substructure by refinement of the sub-particle orientations and origins in a process referred to as ‘localized reconstruction’ [4]. The alignment parameters of the sub-particles can be used to limit the orientation searches around the initial parameters calculated from the orientation of the particle. The orientations of the sub-particles can also be transformed to confer to the standard orientation of the substructure’s local symmetry. In addition, this approach allows integrating localized reconstruction with the symmetry relaxation approach described above [7], for instance to test the five possible rotations for each of the hexamers bound to the 12 icosahedral vertexes.

**Figure 5:**
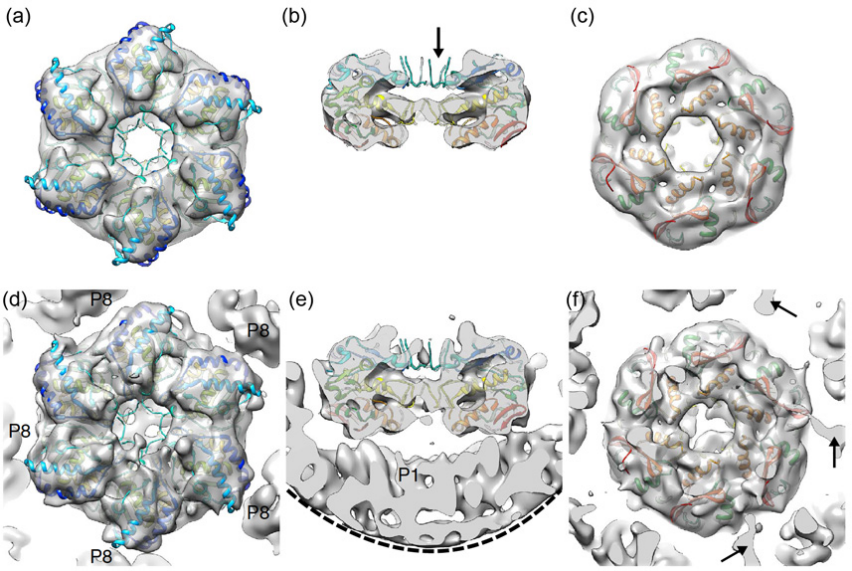
Hexameric packaging NTPase of bacteriophage ϕ6 (**a**–**c**) Localized reconstruction of the hexamer, reconstructed with six-fold symmetry, is shown from the top (a), side (b) and below (c). Atomic model of the hexamer (PDB:4BLO) fitted in the reconstruction is colored from red (C-terminus) to blue (N-terminus). Flexible loops that were unresolved are indicated (arrow). (**d**–**f**) Same views as in (a)–(c) showing the asymmetric localized reconstruction. Five neighboring proteins (P8) around the hexamer are labeled in (d). The edge of the mask cutting through the protein shell (P1) under the hexamer is indicated with a dashed line in (e). Three densities possibly connecting the P1 shell to the C-termini of the hexamer are indicated with arrows in (f). Figure reproduced from [12].

Localized reconstruction has recently been used to reconstruct the hexameric packaging NTPase of the icosahedral bacteriophage ϕ6 bound to all of the 12 five-fold symmetric vertices (12 I-C5–C6 symmetry mismatches per particle; Table 1) [12]. Prior to reconstruction, the density of the icosahedral protein shell was subtracted in the images and the C6 symmetry axes of the hexamers were aligned in the standard orientation (along the Z-axis) allowing reconstructing its structure with C6 symmetry (Figure 5a–c). Finally, the original images containing the icosahedral capsid were used to reconstruct the vertex structure without symmetry, which allowed to partially resolve the connections between the two symmetry-mismatched components (Figure 5d–f).

### Classification of substructures with variable occupancy

Variable occupancy, where one or more binding sites in a complex bind a substructure with less than 100% occupancy, can result in complex mixtures of different types of symmetry-mismatched particles (Figure 1f). In the simplest case, a population of asymmetric structures (C1) that variably bind an asymmetric substructure (C1-C1–*v*C1) consists of only two types of such particles(subunit bound or unbound). In a slightly more complicated case, a particle with the lowest possible actual symmetry, two-fold symmetry (C2), has two asymmetric units, each of which can in theory bind an interacting subunit. In the population of such C2-C1–*v*C1 particles, there are three types of particles (no subunits bound, one subunit bound, or both subunits bound). Such relatively simple cases can be tackled with standard 3D classification approaches where particles are classified in different occupancy states [43]. However, currently the maximum number of classes used in 3D classification is limited by computational considerations to approximately 10–15 [29].

When the dominant symmetry of the complex increases, the number of different occupancy states increases exponentially and it becomes unpractical to separate them by conventional image classification methods. For example, a virus capsid with icosahedral symmetry has 60 asymmetric units, and thus 60 possible binding sites, each of which can be either occupied or unoccupied (I-C1–*v*C1 symmetry mismatch). This leads to a very large number of different occupancy states (in the order of 2^60^) [44]. In such complex cases, sub-particle based localized reconstruction methods become useful [4,5]. Since each sub-particle corresponds to only two possible states (bound or unbound), 3D classification using in principle only two classes can be used to separate them. Similarly, if the substructures have different discrete conformations, they can be separated by classifying the sub-particles [2,4,34,45].

**Figure 6:**
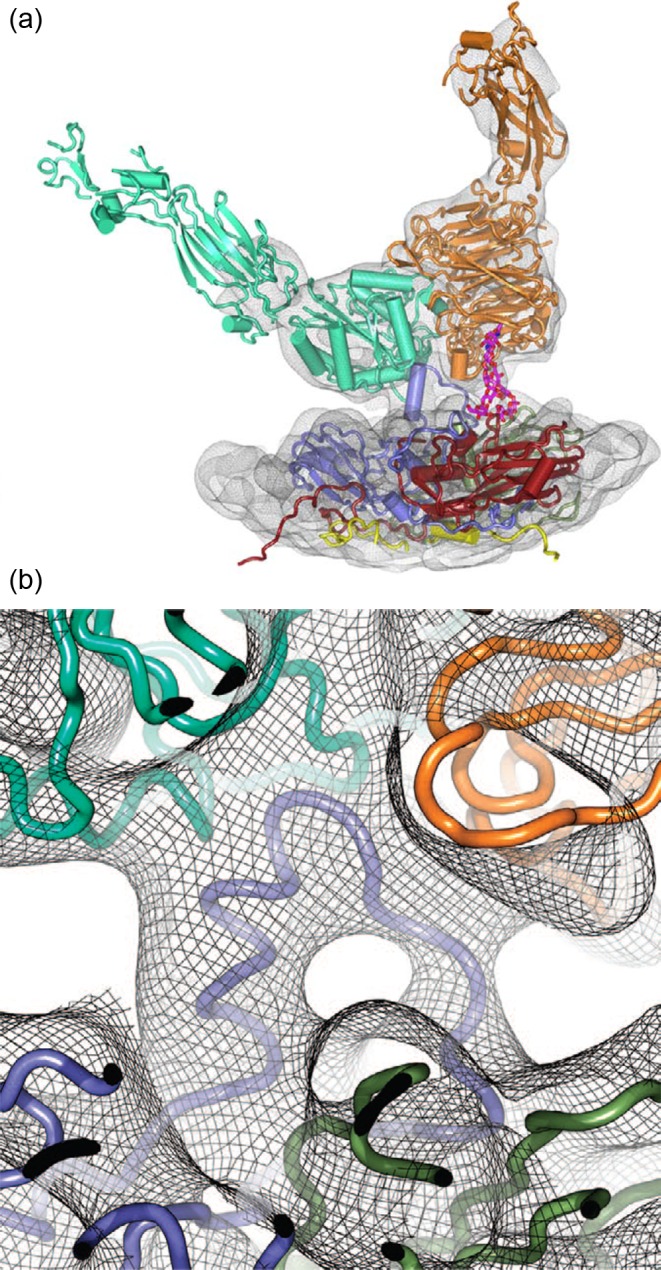
Foot and mouth disease virus binding to integrin receptor (**a**) Localized reconstruction of the integrin molecule (orange and mint) engaged with the binding site on the underlying viral capsid (different capsid proteins colored in blue, red, and yellow). A resolved glycan is in magenta. (**b**) A close-up of the integrin–capsid interaction. The RGD-loop interacting with the integrin is in blue. Reproduced from [5].

Localized reconstruction has been used recently to reconstruct the structure of purified integrin ectodomains bound to asymmetric units of foot and mouth disease virus [5]. 3D classification of different occupancy states and conformations of integrin sub-particles allowed resolving the structure of the integrin bound to the RGD-loop on the viral surface (Figure 6; I-C1–*vf*C1 symmetry mismatch). This method is expected to be widely applicable to address other complexes with substructures showing variable occupancy and different conformations.

### Refinement of subunits with quasi-symmetry or flexibility

Several approaches exist for dealing with structures manifesting inherent flexibility (Figure 1c), such as the ratchet-like motion of the ribosomal subunits [28,29], the quasi-symmetric C8 arrangement (*q*C8) of asymmetric units in the NPC [3], or quasi-octahedral (*q*O) arrangement of asymmetric units in the COPII cages [4,20]. Symmetry expansion/focused refinement [39] and sub-particle based methods [4,37] are well-suited for this task, as they allow breaking the particle into separate asymmetric units, allowing tracking their individual motions and rotations. Contribution of the overlapping asymmetric units can be removed from the images by partial signal subtraction, and this can be iterated by taking into account their movements and rotations established in the previous iteration [39,46]. Refinement of the sub-particle angles may then allow coherent averaging of such flexible subunits [4,39].

**Figure 7:**
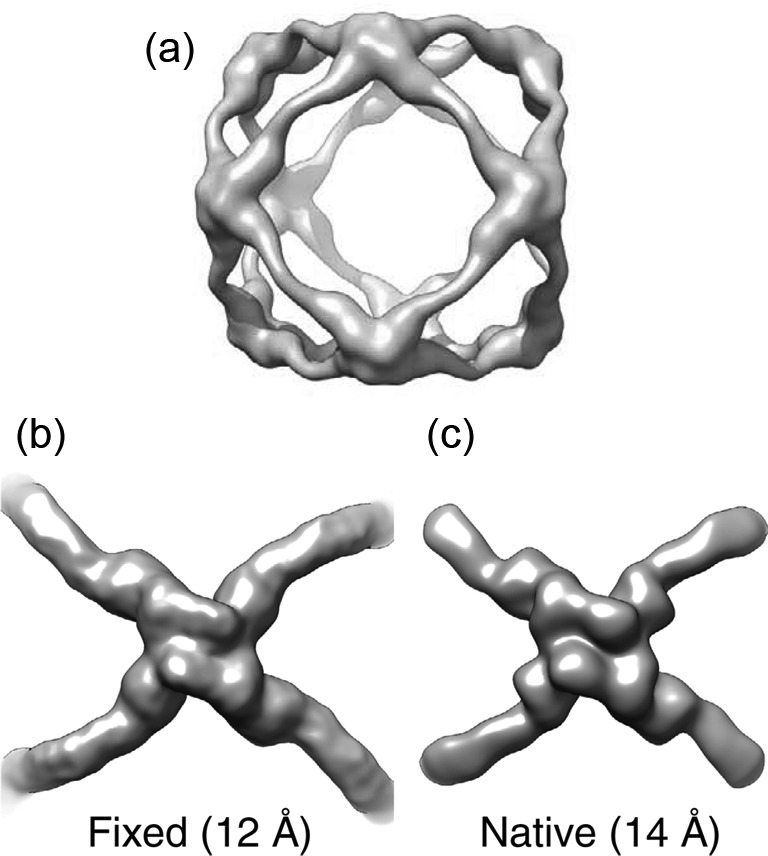
Improving the reconstruction of native COPII cages by localized reconstruction (**a**) Reconstruction of the COPII cage determined by conventional refinement from native particles and assuming octahedral symmetry. Due to flexibility, the resolution is limited to 35 Å [4]. (**b**) One vertex of the COPII cage, extracted from the reconstruction calculated from fixed particles at 12 Å resolution [21] and aligned along its C2 symmetry axis. (**c**) Reconstruction of the vertex, solved by localized reconstruction from the same particles as used in (a). At improved resolution of 14 Å, similar features were resolved as by fixing the particles. Figure reproduced from [4].

Initial reconstructions of COPII cages were limited to ~30 Å resolution due to their flexibility (quasi-octahedral symmetry) [20] and possibly also deformations at the air–water interface [21]. Chemical fixation was subsequently used to increase the stability of these complexes, which allowed to improve the resolution to 12 Å [21]. More recently, a similar resolution gain was achieved by localized reconstruction (Table 1) [4]. Individual vertices of the cages were extracted as sub-particles from images of unfixed cages. Refining the movements and rotations of the vertices allowed reconstructing the vertex structure at 14 Å resolution (Figure 7).

In another study, the structure of Rift Valley fever virus was limited to 13 Å resolution, suggesting that the virus deviates from icosahedral symmetry applied in the reconstruction (quasi-icosahedral symmetry; Table 1). Treating the viral capsomers (12 pentamers, and 60 type 1, 2, and 3 hexamers) as sub-particles in localized reconstruction allowed to improve their resolution to 7.7–8.6 Å [35]. Localized reconstruction is thus expected to be applicable in improving the resolution of different quasi-symmetric structures. However, unless properly treated by partial signal subtraction, overlaps between the sub-particles in these cases may limit the attainable resolution.

## Concluding remarks

Examination of 3D density maps often reveals areas of blurred out density, which may be due to the presence of a symmetry-mismatched component. The methods discussed in this review may allow coherent averaging of the blurred-out densities to better resolve their structures. This in turn, often in combination with other methods, may lead to better understanding of the related biological function.

In this review, I have focused on macromolecular complexes manifesting different types of symmetry mismatches and their combinations, including pseudo- and quasi-symmetry, variable occupancy, and flexible subunits (Figure 1). However, it is not just complexes that suffer from these effects limiting coherent averaging; most, if not all, individual macromolecules can be considered to exhibit some degree of flexibility or conformational heterogeneity in solution [29]. In this light, even asymmetric single particles can be considered to exhibit quasi-C1 ‘symmetry’ when they deviate from the assumed, idealized structure.

In general, flexibility is often continuous and image classification methods that rely on dividing the data in discrete classes are unsuitable for capturing all of the states sampled by the macromolecule [29]. Emerging methods based on the concepts of multi-particle analysis [47], manifold embedding [48], and normal modes analysis [49] are expected to improve the current classification methods. Also how to accurately reconstruct density of the structure after flexibility and symmetry mismatches in the substructures have been removed, remains a topic of active method development. These methods are expected to allow coherent averaging of symmetry-mismatched substructures from many complex macromolecular assemblies at increasing level of detail, ultimately contributing to our understanding of their elaborate functions.

## Abbreviations

cryo-TEM: cryogenic transmission electron microscopy
NPC: nuclear pore complex
